# CT and ultrasound findings of amiodarone-induced epididymitis

**DOI:** 10.1016/j.radcr.2026.07.069

**Published:** 2026-08-13

**Authors:** Akihiko Sakata, Eri Kato, Tomonori Matsushita, Yuki Kita, Takashi Kobayashi, Yuji Nakamoto

**Affiliations:** a Department of Diagnostic Imaging and Nuclear Medicine, Kyoto University Graduate School of Medicine, Kyoto, Japan; b Department of Cardiovascular Medicine, Kyoto University Hospital, Kyoto, Japan; c Department of Urology, Kyoto University Hospital, Kyoto, Japan

**Keywords:** Amiodarone, Epididymitis, Computed tomography

## Abstract

Amiodarone-induced epididymitis is a rare but clinically significant noninfectious complication of long-term amiodarone therapy. It typically presents with bilateral scrotal pain and swelling, with ultrasonography demonstrating epididymal hypervascularity, and symptoms generally resolve upon drug discontinuation. We report the case of a 52-year-old man with left ventricular noncompaction cardiomyopathy who presented with bilateral, predominantly left-sided pain after approximately 4.5 years of amiodarone therapy. Noncontrast computed tomography (CT) of the abdomen and pelvis demonstrated bilateral epididymal hyper attenuation, a finding not previously described in the literature. Given the concurrent presence of hepatic high attenuation consistent with amiodarone deposition, amiodarone-induced epididymitis was suspected, and the drug was discontinued. Symptoms resolved completely following discontinuation. We hypothesize that this CT appearance reflects the local accumulation of iodine-rich amiodarone metabolites within epididymal tissues. Although the specificity of this finding remains unknown, epididymal hyper attenuation on CT may raise the possibility of amiodarone-induced epididymitis in patients receiving the drug, prompting radiologists to suggest this diagnosis and helping to avoid unnecessary antibiotic treatment.

## Introduction

Amiodarone is an iodinated benzofuran derivative and a highly effective antiarrhythmic agent widely used to treat refractory ventricular arrhythmias and atrial fibrillation [1]. Despite its clinical efficacy, amiodarone has an extensive adverse effect profile due to its high lipophilicity and long elimination half-life, leading to drug accumulation in various tissues. While systemic toxicities affecting the liver, lungs, skin, and thyroid are well known, amiodarone-induced epididymitis is a relatively rare and underrecognized noninfectious complication [[2], [3], [4], [5], [6], [7], [8], [9], [10], [11], [12], [13], [14]]. Patients typically present with bilateral scrotal pain and swelling, which generally resolve upon dose reduction or discontinuation of the drug, although symptoms may recur upon rechallenge [9]. Previous imaging reports of this condition have been limited to scrotal ultrasonography (US) and MRI [12], with no CT findings described to date. We herein report a case of amiodarone-induced epididymitis with previously undescribed CT findings.

## Case report

A 52-year-old man with left ventricular noncompaction cardiomyopathy, listed for heart transplantation, presented with a 10-day history of lower abdominal discomfort. Amiodarone had been initiated 4.5 years earlier for heart failure exacerbation precipitated by new-onset atrial fibrillation, and had been continued since then, with a cumulative dose of approximately 390 g and a dose of 400 mg/d at the time of presentation. The pain was bilateral but predominantly left-sided, aggravated by walking and contact with clothing, and partially relieved by loxoprofen. Physical examination revealed tenderness over the left inguinal region. Body temperature could not be reliably assessed because the patient was already taking a nonsteroidal anti-inflammatory drug. Laboratory data showed mildly elevated C-reactive protein (1.34 mg/dL) and known hepatic dysfunction with a cholestatic pattern (aspartate aminotransferase 35 U/L, alanine aminotransferase 90 U/L, alkaline phosphatase 139 U/L [International Federation of Clinical Chemistry and Laboratory Medicine method], gamma-glutamyl transpeptidase 152 U/L). Routine laboratory monitoring during the same episode showed a normal white blood cell count (7820/µL; neutrophils 78.2%), and urinalysis showed no evidence of infection. Urine culture was not performed. The patient reported no recent sexual contact.

**Fig. 1:**
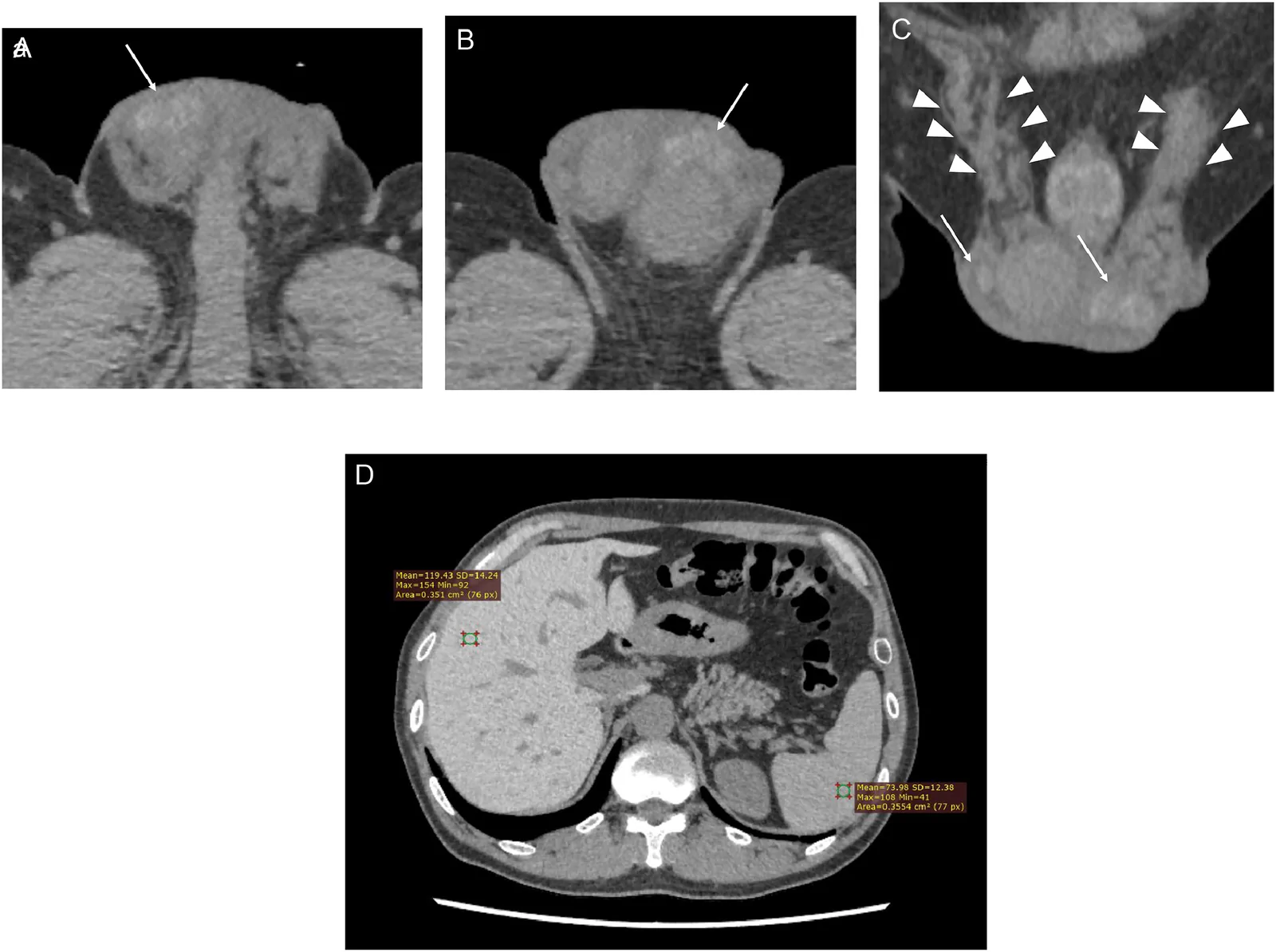
Noncontrast CT of the abdomen and pelvis. (A and B) Axial images show hyper attenuating right (A) and left (B) epididymides (arrows). (C) Coronal image shows hyper attenuation of the bilateral epididymides (arrows) and swelling of the bilateral spermatic cords (arrowheads). (D) Axial image at the level of the liver with regions of interest placed in the liver and spleen; hepatic attenuation (mean 119.4 HU) is increased relative to the spleen (74.0 HU), consistent with amiodarone deposition.

**Fig. 2:**
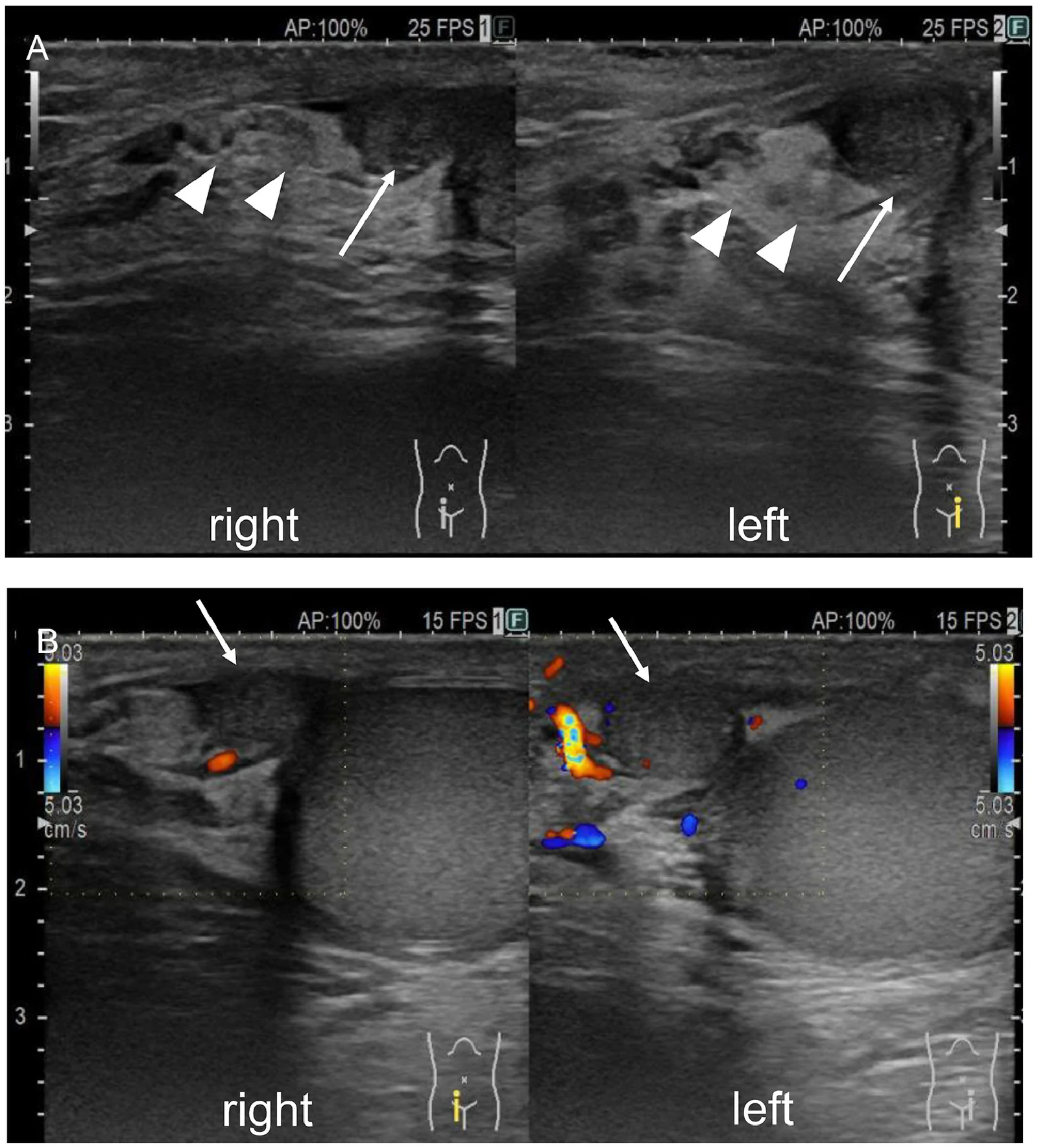
Scrotal ultrasound performed by a urologist; the right and left sides are labelled on the images. (A) B-mode image shows swelling of the spermatic cords (arrowheads) and the epididymides (arrows, 1 on each side). (B) Color Doppler image shows increased vascularity of the epididymides bilaterally (arrows, 1 on each side).

Noncontrast CT of the abdomen and pelvis was performed to evaluate the lower abdominal and inguinal pain and to exclude urinary calculi and inguinal pathology. CT revealed swelling of the bilateral spermatic cords and hyper attenuation of both epididymides (Fig. 1A-C). On region-of-interest measurement, the epididymides showed mean attenuation values of 61.1 Hounsfield units (HU) on the right and 57.7 HU on the left, whereas the testes, which did not appear visually hyperattenuating, measured 47.2 HU and 45.7 HU, respectively (Supplementary Figs. 1 and 2). In addition, hepatic attenuation was increased (mean 119.4 HU, compared with 74.0 HU in the spleen), consistent with amiodarone deposition in the liver (Fig. 1D). The thyroid was outside the scanned field of view, and only the lung bases were partially included, without findings suggestive of amiodarone pulmonary toxicity. Given the bilateral epididymal hyper attenuation together with the hepatic findings and the patient's long-term amiodarone therapy, amiodarone-induced epididymitis was suspected, and the patient was referred to the urology department. Scrotal US, performed and interpreted by a urologist, demonstrated bilateral inflammatory change involving the epididymis and spermatic cord with increased vascularity, and an infectious cause was considered unlikely (Fig. 2). Amiodarone was discontinued and was not resumed. No antibiotic therapy was administered. The pain had resolved completely by the follow-up visit approximately 1 month later. Based on the overall clinical course, a final diagnosis of amiodarone-induced epididymitis was made.

## Discussion

Amiodarone-induced epididymitis is an uncommon adverse effect with a reported incidence of 3%-11%, varying with the dose and duration of therapy. The onset of symptoms is highly variable, typically appearing anywhere from 4 to 71 months after the initiation of amiodarone treatment. Patients characteristically present with chronic epididymal pain (epididymalgia) and swelling, notably in the absence of fever, leukocytosis, or urinary tract symptoms. A characteristic feature is that the involvement is frequently bilateral, although symptoms are often more severe on 1 side, which can initially mask the bilateral nature of the disease. Furthermore, the epididymitis typically improves and resolves within 10 days to 3 months upon reduction of the amiodarone dose or complete discontinuation of the drug, in accordance with its extraordinarily slow elimination rate [10].

As its presentation can mimic bacterial epididymitis or testicular torsion, the diagnosis fundamentally requires careful exclusion of other causes of acute or chronic scrotal pain. In this regard, scrotal US is the most frequently utilized and reported modality. Doppler US typically reveals non-specific inflammatory changes, including unilateral or bilateral epididymal enlargement, hyperemia, and occasional reactive hydroceles. Additionally, MRI has been utilized in limited cases, demonstrating marked epididymal edema on T2-weighted images and significant contrast enhancement [12]. Unfortunately, both US and MRI findings are largely indistinguishable from those of infectious epididymitis. Nevertheless, clinical clues such as bilateral involvement, absence of infectious signs, and a history of amiodarone use can raise suspicion for this drug-induced etiology.

To the best of our knowledge, this is the first case to report a high-attenuation area in the epididymis on CT scan. The mechanism behind this radiological appearance can be explained by the pharmacological properties of the drug. Amiodarone and its major metabolite, desethylamiodarone, accumulate markedly in genital tissue. In necropsy specimens, testicular concentrations have been reported at levels several thousand-fold higher than those in plasma [15], and epididymal tissue concentrations exceeding 400 times the upper limit of normal have been documented in an affected patient [2]. Consistent with this, the seminal concentration of desethylamiodarone in a patient with amiodarone-associated epididymitis was approximately 5 times the corresponding serum concentration [4]. Previous histological examinations of affected epididymides have confirmed the presence of focal fibrosis, lymphocytic infiltration, and distinctive phagocytosis of amiodarone-metabolite crystals by histiocytes [11]. Besides the epididymides, we also measured the testes in our patient (47.2 and 45.7 HU). These values cannot be interpreted as elevated in the absence of reference data, but they are not inconsistent with the concomitant gonadal deposition suggested by the pharmacological literature. It is well established that substantial amiodarone deposition in organs such as the liver and lungs results in high-attenuation areas on CT [16,17]. Extrapolating from this established phenomenon, we hypothesize that the local accumulation of iodine-containing amiodarone metabolites in the epididymis may account for the high-attenuation appearance observed on CT in our case. Histological confirmation was not obtained, and this proposed mechanism therefore remains inferential. It should be emphasized that no reference values exist for epididymal or testicular attenuation on noncontrast CT. The values reported here are therefore descriptive, and we do not treat the difference between the epididymis and the testis as diagnostic evidence; the quantitative anchor for systemic iodine deposition in this case is the hepatic attenuation relative to the spleen.

The bilateral distribution of the findings deserves emphasis, as it carries considerable diagnostic weight. Bacterial epididymitis arises almost exclusively from retrograde ascent of pathogens along the vas deferens and is therefore virtually always unilateral. In contrast, amiodarone-induced epididymitis results from systemic accumulation of the drug in tissue, and bilateral involvement is characteristic; in the original case series, all 6 affected patients ultimately had bilateral involvement, although 5 initially presented with unilateral enlargement that became bilateral within 3 weeks, and no infectious etiology was identified in any of them [2]. This contrast is grounded in the underlying mechanism rather than in empirical observation alone, and bilateral involvement therefore constitutes strong evidence against an infectious cause. In our patient, bilateral involvement was demonstrated concordantly on CT and US, and was supported by the absence of leukocytosis, a negative urinalysis, the absence of recent sexual contact, and resolution of symptoms without antibiotic therapy.

Paradoxically, this diagnostically valuable finding is the one least likely to be appreciated at the bedside. Although the disease is bilateral, symptoms are typically more severe on 1 side, and the discomfort is often localized to the inguinal region rather than the scrotum, so that the condition may be perceived as a unilateral problem. Moreover, patients receiving amiodarone are primarily managed by cardiologists or general practitioners for severe underlying arrhythmias, and scrotal symptoms may not be readily attributed to the drug in these settings. Our case followed precisely this course: the initial assessment localized the pain to the left inguinal region, and CT was performed to evaluate lower abdominal and inguinal pain. It was CT that first demonstrated the bilateral epididymal and spermatic cord abnormality, together with hepatic hyper attenuation indicating systemic iodine deposition, and this prompted urological referral, after which US confirmed bilateral inflammatory change. Because cross-sectional imaging evaluates both sides irrespective of the laterality of symptoms, radiologists interpreting abdominal and pelvic CT in patients receiving amiodarone are well positioned to suggest this diagnosis before the drug has been identified as the cause, and to help avoid unnecessary antibiotic therapy. Accumulation of further cases will be needed to characterize the CT appearance of this entity more fully.

This report has several limitations. As noted above, the proposed mechanism was not confirmed histologically. No reference data exist for the attenuation of the normal or abnormal epididymis on noncontrast CT, and we could not identify any prior study reporting such values; the specificity of epididymal hyper attenuation is therefore unknown, and other conditions, such as granulomatous epididymitis, might conceivably produce a similar appearance. Accordingly, we do not propose this finding as specific to amiodarone, but rather as a clue that may raise the possibility of amiodarone-induced epididymitis in a patient known to be receiving the drug, particularly when accompanied by hepatic hyper attenuation and bilateral involvement. Follow-up imaging after drug discontinuation was not obtained, so the reversibility of the CT findings could not be assessed. As amiodarone was not readministered, a contribution of the natural course to symptom resolution cannot be entirely excluded. Finally, the available ultrasound images demonstrate the described findings less clearly than would be ideal, and epididymal dimensions could not be reliably measured retrospectively; the bilateral involvement therefore rests on the urologist’s assessment at the time of scanning together with the CT findings, rather than on the ultrasound images alone.

## Declaration of generative AI and AI-assisted technologies in the manuscript preparation process

During the preparation of this work the author(s) used Claude (Anthropic) in order to assist with English language editing and refinement of the manuscript. After using this tool/service, the author(s) reviewed and edited the content as needed and take(s) full responsibility for the content of the published article.

## Patient consent

Written informed consent was obtained from the patient for publication of this case report and accompanying images.

## Appendix Supplementary materials

Supplementary Fig. 1 – Region-of-interest measurement of the right epididymis on non-contrast CT. The mean attenuation value was 61.1 HU. Measurements were performed using RadiAnt DICOM Viewer 2025.1 (Medixant, Poznań, Poland). Image, image 2 dummy alt text

Supplementary Fig. 2 – Region-of-interest measurements of the testes and the left epididymis on non-contrast CT. Mean attenuation values were 57.7 HU for the left epididymis, 47.2 HU for the right testis, and 45.7 HU for the left testis. Measurements were performed using RadiAnt DICOM Viewer 2025.1 (Medixant, Poznań, Poland). Image, image 1 dummy alt text
